# Presenting prevalence and management of psychosocial problems in primary care in Flanders

**DOI:** 10.1186/s13690-015-0061-4

**Published:** 2015-03-09

**Authors:** Lena Vannieuwenborg, Frank Buntinx, Jan De Lepeleire

**Affiliations:** Department of General Practice, KU Leuven, Kapucijnenvoer 33 blok j - bus 7001, 3000 Leuven, Belgium

**Keywords:** Primary care, Interdisciplinary Work, Epidemiology, Psychosocial problems

## Abstract

**Background:**

Psychosocial problems are widespread but reliable data about management are sparse. An overall view is missing and there is a need for a wider framework to include the data available in health care and welfare practice, databases and research output. The question under scope is: how are psychosocial problems presented and handled in primary care in Flanders?

**Methods:**

A mixed method was used. Using a ‘fishbone diagram’ (1) we obtained a basic structure to visualize the main (problem) areas and challenges. A literature study (2) and semi-structured interviews with health care and welfare professionals in primary care (3) were performed. Finally, two interdisciplinary focus groups were organized (4).

**Results:**

In Flanders, there is no tradition of multidisciplinary psychosocial research in primary care causing a lack of integrated data. Data only exist within disciplines without transdisciplinarity. The data are difficult to interpret due to different labeling and registration processes between disciplines and settings. However, we can find some general trends: assistance to patients with psychosocial problems is based on what can be offered, rather than on patient needs; drug treatment remains popular; referral of patients within primary care or to secondary care does not seem to be obvious. Among all disciplines, there is a great need for more collaboration and considerable advantages are to be expected from the growing emergence of multidisciplinary practices; multiculturalism appears to take an increasingly important place within primary care in Flanders and has implications for the care offered; and treatment effectiveness in psychosocial problems seems to be more related to the person of the caregiver than to a specific discipline, theory or type of treatment.

**Conclusions:**

Based on our results, we strongly advise stimulation and organization of integrated (multidisciplinary) research regarding psychosocial problems in primary care and a more consistent registration by the agencies in primary care.

## Background

The prevalence of mental health problems in Flanders is high, as shown in figures from Intego, a Flemish database that registers and analyzes the prevalence of illness as presented to Flemish general practitioners. An extrapolation based on these figures shows that in one of the pilot regions with 560,000 inhabitants and about 500 full-time equivalent general practitioners, approximately 75,000 people suffer from one or more mental health problems [1]. These include about 11,000 depressions, 3,000 acute stress reactions, 3,000 severe sleeping problems, 1,500 cases of burn-out, etc. A rough inventory in our research group at the Department of General Practice in Leuven showed that ample data were available, but neither easy to retrieve, nor to handle.

In spite of the available data, a general overview of issues that could be of use to health care and welfare practices, databases and research projects is lacking. The purpose of our study was to identify, collect and interpret available data from various disciplines and settings in Flemish Primary Health Care, and to describe experiences and current needs prevailing in practice [2].

We used the Goldberg and Huxley’s filter model representing five levels of care and accompanying ‘filters’ between them [3,4]. These filters relate to the behavior of patients and care providers. The first three levels are of interest for us, since they refer to primary care: (1) Community (filter: decision to consult a primary care provider), (2) Primary care (filter: recognition of the problem by the primary care provider), and (3) Primary care (filter: referral to secondary care) [3-5].

Based on these three levels, we divided our main research question into three subquestions: 1. How do psychosocial problems present in primary care in Flanders? 2. How are these problems approached? 3. How is further care provided?

## Methods

Between October 2012 and April 2013, we first developed a basic structure to visualise the main (problem) areas and challenges using a ‘fishbone diagram’ [6,7]. Second, we reviewed the literature using PubMed, PsycINFO, Lirias, LibriSource+, Libis, Embase, Google, Google Scholar, Google Books, psychiatric, psychological and medical journals, and (research) data and databases available within the organisations we approached. Thirdly, 21 semi-structured interviews were performed with health care and welfare professionals in primary care. Predetermined topics (derived from the fishbone diagram and literature) formed the guideline for the interviews as to explore the thoughts and experiences of the interviewee with the various items. As the interviewee determines the course and content of the conversation in semi-structured interviews, the interviewer followed the discourse of the interviewee as much as possible, while simultaneously ensuring that the predetermined topics and questions were discussed during the interview. Depending on the practical context, some interviews were held face-to-face (n = 11), others by telephone (n = 8) and some by mail (n = 2). The latter were sent as structured interviews, but space was provided to give the interviewee the opportunity to express possible thoughts and ideas that were not yet addressed in the questions. Finally, two interdisciplinary focus groups were organized, one in November 2012, one in January 2013, each lasting about 2 hours, and consisting of 6 and 7 participants, respectively. We recruited a wide group of primary care workers from five different disciplines: general practitioners, primary care psychologists and –psychiatrists, social workers, and (home) nurses. These groups were chosen so as to include all professional groups involved in psychosocial primary care, and that consequently should implement and should work with any subsequent (practice) changes [8-13].

In view of representativeness, we sought to ensure as much heterogeneity as possible in terms of age, sex, experience, and working areas within the interviews and focus groups.

## Results

### Conceptualization of psychosocial problems

An important finding was that the concept of psychosocial problems is poorly defined and therefore difficult to operationalize. Both literature and health care professionals use vague concepts. There is no consensus on definitions:

1. In literature as well as in our focus groups and interviews with professional care providers, we noted disagreement whether or not to include somatic components of a psychosocial complaint in the definition. Some authors or care providers define somatic elements within the ‘medical-somatic’ field. However, patient complaints do not always allow for a strict division between ‘psychological’ and ‘somatic’ problems. Strictly medical problems will usually also have consequences for the patient’s physical, psychological, as well as social functioning, and vice versa [14].
2. In literature, there is also disagreement about whether or not to categorize psychiatric diagnoses under the ‘psychosocial’ heading [14,15]. Some authors claim psychiatric disorders do not belong in the category of psychosocial problems because of their biological component and/or because psychiatric problems nearly always cause psychosocial problems. Psychosocial problems are thus interpreted as resulting from psychiatric disorders, rather than being part of them. Others disagree with this statement.
3. In daily practice, psychosocial diagnostic labels often reveal more about the way a caregiver intervenes than about the diagnosis of the patient. For example, caregivers sometimes use different terms for the same problem over time, depending on treatment success following initial diagnosis. Even the definition of ‘a problem’ was not straightforward. For this study, a psychosocial problem was defined as a problem for which professional care was sought and which was labelled as a mental health problem and/or a social problem by one of the partners (caregiver or patient/client).

### Operational definition

We here propose an operational definition of psychosocial problems, based primarily on the literature and interviews with individual professional caregivers. This definition was presented at the beginning of both focus groups and further refined based on the resulting feedback and discussions:

*Psychosocial problems include the broad spectrum of all complaints which are not strictly medical or somatic. They affect the patient’s functioning in daily life, his or her environment and/or life events.*

*On the one hand, it concerns various psychological problems such as: anxiousness, nervousness, tenseness, (posttraumatic or acute) stress, depression and feeling depressed, burn out, loneliness, irritability, sleep disorder, sexual problems, tics, alcohol abuse, tobacco abuse, drug abuse, memory problems, behavior problems, learning difficulties, phase-of-life problems, fear of mental illness, psychoses, schizophrenia, anxiety(disorder), somatization disorder, suicide/suicidality, neurasthenia/surmenage, phobia/obsessive compulsive disorder, personality disorder or identity problem, hyperkinetic disorder, intellectual disabilities, relational problems (with friend, family and/or partner), medically unexplained symptoms and eating disorders.*

*On the other hand, it concerns various social problems such as: poverty/financial problems, housing problems, lack of adequate nutrition or water, social-cultural problems, problems with work or unemployment, school problems, problems with social security, with health care, legal problems, adjustment problems, loss/death of family/partner and educational problems.*

### Inventory and collection of data

Objective and interpretable data were difficult to find, in literature as well as in daily practice-based registrations or research results. In non-medical disciplines (psychologists, nurses, social workers) the problem is even more apparent. For instance, Belgian primary care psychologists are not officially recognized as a licensed profession, which means research or registration are virtually absent. Furthermore, because psychological care costs are not reimbursed, there is no registration by health insurance agencies of psychologist interventions.

Data acquisition and registration pose additional problems. Procedures for registration and processing of the available data are neither similar across the various disciplines, nor within the separate instances and settings. Especially overall data on contacts with welfare workers (social work, home nursing) for psychosocial problems were difficult to find. In Belgium or Flanders there is no general, unambiguous registration and/or processing by the combined welfare agencies. Therefore national or regional data are unavailable. However, cities and towns do provide data. Data structure and registration mode show strong regional differences, which makes comparisons difficult. Moreover, registration possibilities by care professionals frequently appear to be limited. In some health insurance instances the number of categories with regard to a type of contact is limited to two. Hence, counselors will prefer to register the practical aspects of the consultation (arrangement of papers and documents,…) rather than any psychosocial aspects (emotional support,…).

### Presentation of psychosocial problems in primary care in Flanders

Flemish primary care does not have a tradition of multidisciplinary psychosocial research. Integrated data concerning psychosocial problems across the different primary care disciplines are missing.

Furthermore, the same psychosocial problem may receive different labeling and/or registration according to discipline or setting. Patient or social factors can contribute to this, as one problem is sometimes presented differently by the patient/client to different care givers or disciplines, and may therefore also be labeled or registered under a different name. Some possible reasons for this shortcoming can be:

- *Proto-professionalization* [16,17]. Patients formulate their problem or their request for help differently depending on the nature of the caregiver consulted. This can result in different labels by different caregivers for the same problem.
- *Patient attributional style* [18-21]. Patients usually attribute their psychosocial problems to certain factors, e.g. to external or situational factors (e.g. marital problems), or to psychological causes (e.g. depression). Different studies show that patients with mental health problems often present their problems somatically and/or often do not bring up psychological or social components themselves.
- *(Self*-)stigma. Many people still seem to have difficulties admitting or indicating that they are struggling with mental health or financial problems. Other research also reports psychiatric patients continues to be stigmatized, even among professional caregivers [22].

### General practitioner (GP) as an important gateway to primary care

The GP appears to be an important gateway to primary care for patients with psychosocial problems, illustrated by the fact that GPs appear to be involved in 60-80% of consultations for emotional problems [23,24]. Moreover, they appear to be the first caregivers to be consulted and the majority of these cases remains with their GP for follow-up even after being referred [25]. Especially for socially disadvantaged groups (poorly educated and people with lower income), the GP acts as a driving force in addressing mental health problems [26].

Within our focus groups and interviews, it was noted that the medical mandate of the GP may break down barriers, since patients do not feel under any pressure to bring up possible social or psychological problems with their GP. However, this can also become a disadvantage, particularly when a patient continues to focus too much on his medical-somatic problems and shows little or no openness to discuss the psychological components of his complaint(s).

### Management of psychosocial problems in primary care in Flanders

In line with our findings concerning data on prevalence and presentation of psychosocial problems in primary care, we found that data on the approach of the (primary care) psychologist- and psychiatrist, social workers and nurses are sparse and less elaborate compared to data on the approach of GPs. From family medicine, a lot of specific research is available with both international and specifically Flemish data [18,27-31]. However, there is a lack of more general, reliable and easily interpretable data on the management and care supply of other disciplines.

A number of factors can play a role in this. Data on the management by psychiatrists often focus on secondary care, even though they are often also involved in primary care. Data from welfare are geographically spread and registered differently by (different branches of) authorities. These factors prevent an overall view of primary care interventions for psychosocial problems.

### General trends

Despite these limitations we can derive some general trends from research available in Flanders, our interviews, and the focus groups.

### Supply versus need

Based on the information that we obtained from the interviews and focus groups we notice that assistance to patients with psychosocial problems still seems to be overly ‘supply-driven’. Assistance is based on what can be offered, rather than on patient needs.

### Drug treatment remains popular

Based on our review of the literature and research available in Flanders, we find that a pharmacological approach remains popular in the treatment of people with mental health problems, whether or not in combination with non-pharmacological treatment. Also, many patients who do not meet the criteria for a mental disorder, but are treated for emotional problems, are prescribed medication [23].

Furthermore, data on the use of psychotropic drugs show an increase in the already frequent use of antidepressants, stimulants, and antipsychotics [1,32]. The use of tranquillizers and sleep medication remains constant [1]. Despite recent efforts to reduce the use of these potentially addictive products, there is an increase in the prescription of antidepressants in Flanders compared to 10-15ys ago. Moreover, this increase does not match an increase in the number of diagnosed depressions [33]. The chronic use of this medication once prescribed, as well as the prescription of antidepressants for other conditions and problems (for example chronic pain, sleeping problems in the elderly, etc.) could be possible explanations.

### Referral of patients within primary care or to secondary care

There are some considerations concerning referrals for psychosocial problems. GPs help 90% of the patients with psychosocial problems themselves [28]. GPs offer psycho-education and (psychotherapeutic) counseling, but in many studies this frequently used approach is not clearly recorded as opposed to the pharmacological approach that may also be used.

Referral for psychotherapy does not seem to be obvious. It is a time-consuming process, often spread over time, which needs to be run through together with the patient [34]. Patients do not always (immediately) agree with advice for referral. Associated financial implications or stigma may constitute a barrier for the patient. Having a psychologist working in the general practice appears to lower the threshold for referring patients [35]. Among all the different disciplines, there is a great need for more collaboration and considerable advantages are to be expected from the growing emergence of multidisciplinary practices.

Based on the experiences of interviewed professionals, we noted that the process of referral to secondary care was sometimes hampered because of various reasons. Patients may refuse because they have multiple problems (practical, financial, stigma,…). Furthermore, there are usually long waiting lists. Finally, intake procedures can impede the course of care. Patients sometimes have to talk to a number of caregivers, on different occasions, before they are finally referred to a permanent caregiver for further treatment. This can lead to a decrease in the patient motivation or to non-compliance.

GPs referring patients may lose sight of the patient’s further development. The patient is followed up elsewhere and does not always return to the GP. A possible explanation given by professionals in the focus groups is the fact that once a patient enters psychiatry or a specific care project, he is not referred back to the GP soon enough and/or patients are held in psychiatric care for too long, also when this is no longer required. Specific advice from psychotherapeutic care about subsequent treatment of the patient by the GP or other care providers is very rare.

### The aspect of multiculturality

Based on the information from the interviews and focus groups, we notice that multiculturalism appears to take an increasingly important place within primary care. It does not only involve different countries of origin, but also important socio-demographic differences (specific care of the elderly or youth, underprivileged populations, etc.). Different cultures have different languages, understandings, reactions, expectations of care, support systems, etc. The current care offered may not be sufficiently and/or appropriately adapted to these differences. Hence, caregivers encounter difficulties. We noticed growing frustration among caregivers and some were in danger of losing interest or of giving up.

### The person of the caregiver

Treatment effectiveness in psychosocial problems seems to more related to the person of the caregiver than to a specific discipline, theory or type of treatment. For example, a psychotherapeutic approach by the GP may have the same effect as drug treatment in patients with depression. In literature as well as within our focus group sessions we also found that short-term therapy or psychological interventions in patients with depression can be carried out not only by psychotherapists, but also by GPs or nurses without loss of efficacy [36-39].

Different caregivers mentioned that non-specific elements (such as consolidation, acceptance, containment, explanation, a place to ‘speak out’,..) seem to be of particular importance in the treatment of psychosocial problems. These non-specific elements are cross-disciplinary and were already described more than fifty years ago [40]. Especially important seems to be a good personality match between caregiver and patient.

## Discussion

Based on our findings, we would like to highlight some points of consideration towards future course of care and approach to care when faced with psychosocial problems in primary care.

### Multidisciplinary collaboration and access to psychiatric help

More multidisciplinary collaboration between all disciplines involved is necessary. Especially within the family and home care, social workers and nurses experience a great need for training, information, and explanation by mental health care professionals. Partly due to the recent decrease of residential beds in psychiatric hospitals, primary care workers have faced more patients with mental health care problems, for which they are not always adequately trained. Additionally, lack of information exchange from secondary care or other authorities about patients complicates the situation.

The need for access to psychiatric help increases in crisis situations. Recent initial initiatives in this field have been warmly welcomed. However, the need for help on the spot in acute situations (‘red phone’) remains high, for example when a decision has to be taken about whether or not a patient needs to be admitted against his will.

### Some considerations and limitations regarding the course of care

A number of practical considerations and limitations in the care program sometimes appear to be differently experienced by caregivers than by the patient [41,42]. Caregivers sometimes tend to have incorrect ideas about patient experiences, wishes and expectations, without questioning the patient about their reality. This might include, e.g., the financial cost of referral, time needed, resistance to further consultation with a specialized caregiver or to trying psychotherapy. It is possible that thereby patients sometimes are referred to more specialized help too late or too slowly, or that they wrongfully receive only drug treatment, while they may have benefited more from referral or non-pharmacological approach.

Another point of consideration is the prevailing uncertainty among professionals in primary care about their signaling function and –operationalization [43]. When caregivers notice societal problems in patients or families, they currently do not know how to proceed or to whom to report. To date, there no formal procedures or verified care paths to address this.

Finally, we noted that digitalization and professionalization of society creates a financial and digital gap, which affects the weakest and poorest members of society first. Patients in this group do not find their way to help, nor do they know their rights or how to obtain them.

## Conclusions

In Flanders, there is no tradition of multidisciplinary research in primary care, causing a lack of integrated data on psychosocial problems across different disciplines.

Data almost only exist within disciplines, and there is no transdisciplinarity. On the one hand, data are difficult to interpret due to unclear and ambiguous definition of psychosocial problems in literature, as well as in practice. On the other hand, interpretation difficulties are also due to the different labelling and registration processes between disciplines and settings.

In case of psychosocial problems, the GP is a very important gateway to primary care. Furthermore, the person of the caregiver appears to be important for effective treatment, rather than a certain discipline or theory.

Finally, we identified a large need and demand for more and better collaboration, communication and coordination between the actors involved in health care and welfare.

Overall, we strongly advise stimulation and organisation of integrated (multidisciplinary) research regarding psychosocial problems in primary care in Flanders.

## Abbreviations

GP: General practitioner

## References

[1] Department of general practice, KU Leuven. Intego-project. [http://www.intego.be]

[2] Vannieuwenborg L, Buntinx F, De Lepeleire J (2013). Onderzoeksrapport: voorkomen en aanpak van psychosociale problemen in eerste lijn.

[3] Goldberg D (2003). Psychiatry and primary care. World Psychiatry.

[4] Goldberg D, Huxley P (1992). Common mental disorders. A bio-social model.

[5] Verhaak PF (1995). Determinants of the help-seeking process: Goldberg and Huxley’s first level and first filter. Psychol Med.

[6] Hewitt-Taylor J (2012). Identifying, analyzing and solving problems in practice. Nurs Stand.

[7] Ishikawa K (1976). Guide to quality control.

[8] Kitzinger J (1994). The methodology of Focus Groups: the importance of interaction between research participants. Sociol Health Illn.

[9] Kitzinger J (1995). Qualitative Research. Introducing focus groups. BMJ.

[10] Morgan DL (1996). Focus Groups. Annu Rev Sociol.

[11] Morgan DL (1997). Focus Groups as Qualitative Research.

[12] Powell RA, Single M (1996). Methodology Matters - V. Focus Groups. Int J Qual Health Care.

[13] Wollersheim H, Bakker PJM, Bijnen AB, Gouma DJ, Wagner C, van der Weijden T (2011). Kwaliteit en veiligheid in patiëntenzorg.

[14] Joosten AR (1997). Psychosociale verklaringen voor klachten in huisarts-patiëntgesprekken. Een gespreksanalytische studie.

[15] Postma S (2008). JZG-richtlijn. Vroegsignalering van psychosociale problemen.

[16] Alberts JF, Sanderman R, Gerstenbluth I, van den Heuvel WJ (1998). Sociocultural variations in help-seeking behavior for everyday symptoms and chronic disorders. Health Policy.

[17] de Swaan A, van Gelderen R, Kense V (1979). Sociologie van de psychotherapie, vol 2: Het spreekuur als opgave.

[18] Buntinx F, De Lepeleire J, Heyrman J, Fischler B, Vander Mijnsbrugge D, van den Akker M (2004). Diagnosing depression: what’s in a name?. Eur J Gen Pract.

[19] Sigel P, Leiper R (2004). GP views of their management and referral of psychological problems: a qualitative study. Psychol Psychother Theory Res Pract.

[20] Van der Heyden J (2010). Gezondheidsenquête 2008. Contacten met de huisarts. Report.

[21] Zantinge E (2009). Drukke huisartsen houden oog voor psychische problemen. Huisarts Wet.

[22] Adriaensen K, Pieters G, De Lepeleire J (2011). Stigmatisering van psychiatrische patiënten door huisartsen en studenten geneeskunde : een literatuuronderzoek. Tijdschr Psychiatr.

[23] Bruffaerts R, Bonnewyn A, Van Oyen H, Demarest S, Demyttenaere K (2004). Zorggebruik voor mentale stoornissen in Belgie. Resultaten van de European Study on Epidemiology of Mental Disorders (ESEMeD). Tijdschr Geneeskd.

[24] Vanderleyden L, Callens M, Noppe J (2009). De sociale staat van Vlaanderen 2009.

[25] Kovess-Masféty V, Saragoussi D, Sevilla-Dedieu C, Gilbert F, Suchocka A, Arveiller N (2007). What makes people decide who to turn to when faced with a mental health problem? Results from a French survey. BMC Public Health.

[26] Gouwy A, Christiaens W, Bracke P (2008). Mental health services use in the general Belgian population: estimating the impact of mental health and social determinants. Arch Public Health.

[27] Boffin N, Bossuyt N, Declercq T, Vanthomme K, Van Casteren V (2012). Incidence, patient characteristics and treatment initiated for GP-diagnosed depression in general practice: results of a 1-year nationwide surveillance study. Fam Pract.

[28] Fleming DM (1993). The European study of referrals from primary to secondary care. PhD thesis.

[29] Heyrman J, Declercq T, Rogiers R, Pas L, Michels J, Goetinck M (2008). Depressie bij volwassenen: aanpak door de huisarts. Huisarts Nu.

[30] Verhaak PFM, van Weel C (1986). Interpretatie en behandeling van psychosociale klachten in de huisartspraktijk. Ned Tijdschr Geneeskd.

[31] Verhaak P, Tijhuis MAR (1992). Psychosocial problems in primary care: some results from the dutch national study of morbidity and interventions in general practice. Soc Sci Med.

[32] Gisle L, Van der Heyden J, Gisle L, Demarest S, Drieskens S, Hesse E, Tafforeau J (2010). Mentale Gezondheid. Gezondheidsenquête België, 2008. Rapport I - Gezondheidstoestand.

[33] Truyers C, Bartholomeeusen S, De Lepeleire J, Buntinx F (2012). De stijging van antidepressivagebruik in de eerste lijn. Report.

[34] Heyrman J, De Lepeleire J, Heyrman J (2012). Verwijzen voor psychische zorg. Competenties in moeilijke situaties. Over kwaliteit van zorg en communicatie.

[35] Cape J, Parham A (1998). Relationship between practice counselling and referral to outpatient psychiatry and clinical psychology. Br J Gen Pract.

[36] Mynors-wallis LM, Gath DH, Day A, Baker F (2000). Randomised controlled trial of problem solving treatment, antidepressant medication, and combined treatment for major depression in primary care. BMJ.

[37] Mynors-wallis LM, Gath DH, Lloyt-Thomas AR, Tomlinson D (1995). Randomised controlled trial comparing problem solving treatment with amitriptyline and placebo for major depression in primary care. BMJ.

[38] Rogiers R (2002). Unipolaire depressie: niet-medicamenteuze aanpak door de huisarts. Tijdschr Geneeskd.

[39] Van Schaik DJF, van Marwijk HWJ, Van Der Windt DAWM, Beekman ATF, De Haan M, Van Dyck R (2002). De effectiviteit van psychotherapie in de eerste lijn bij patiënten met een depressieve stoornis. Een systematisch overzicht. Tijdschr Psychiatr.

[40] Frank JD, Frank JB (1961). Persuasion and healing. A comparative study of psychotherapy.

[41] Anthierens S, Pasteels I, Habraken H, Steinberg P, Declercq T, Christiaens T (2010). Barriers to nonpharmacologic treatments for stress, anxiety, and insomnia: family physcicians’ attitudes toward benzodiazepine prescribing. Can Fam Physician.

[42] Anthierens S, Habraken H, Petrovic M, Christiaens T (2007). The lesser evil? Initiating a benzodiazepine prescription in general practice: a qualitative study on GPs’ perspectives. Scand J Prim Health Care.

[43] van Meer N, Truyen J, Gorissen H, Mekers G, Buntinx F (2012). Het gebruik van observatieschalen bij tekenen van mogelijke cognitieve problemen thuis. Tijdschr Geneeskd.

